# Physical Versus Virtual Reality–Based Calm Rooms for Psychiatric Inpatients: Quasi-Randomized Trial

**DOI:** 10.2196/42365

**Published:** 2023-05-19

**Authors:** Maria Ilioudi, Philip Lindner, Lilas Ali, Sara Wallström, Almira Osmanovic Thunström, Michael Ioannou, Nicole Anving, Viktor Johansson, William Hamilton, Örjan Falk, Steinn Steingrimsson

**Affiliations:** 1 Psychiatric Department Sahlgrenska University Hospital Region Västra Götaland Gothenburg Sweden; 2 Institute of Health Care Sciences, Centre for Person-Centred Care, Sahlgrenska Academy, University of Gothenburg Gothenburg Sweden; 3 Centre for Psychiatry Research, Department of Clinical Neuroscience, Karolinska Institutet and Stockholm Health Care Services Stockholm Sweden; 4 University of Gothenburg Centre for Person-Centred Care Gothenburg Sweden; 5 Region Västragötaland, Forensic Psychiatric Department, Sahlgrenska University Hospital Gothenburg Sweden; 6 Section of Psychiatry and Neurochemistry, Institute of Neuroscience and Physiology, Sahlgrenska Academy, University of Gothenburg Gothenburg Sweden; 7 Mediary Stockholm Sweden

**Keywords:** psychiatry, psychiatric inpatient care, relaxation, sensory room, virtual reality

## Abstract

**Background:**

Interest in sensory rooms or so-called “calm rooms” in psychiatric inpatient care has increased significantly. In a hospital setting, their purpose is to introduce a relaxing environment to increase well-being as well as to decrease anxiety and aggressive behaviors. Calm rooms can also be used as a tool to provide self-help through a convenient environment for the patients and, at the same time, strengthen the therapeutic relationship between the patient and the professional. Recent developments in virtual reality (VR) have made virtual calm rooms possible, but these have not yet been evaluated in psychiatric inpatient care.

**Objective:**

This study aimed to compare the effects of VR and physical calm rooms on self-reported well-being and physiological markers of arousal.

**Methods:**

The study was conducted in 2 inpatient psychiatric wards specializing in bipolar disorder from March 2019 to February 2021. Patients who were already admitted were asked if they were interested in using a calm room and willing to provide ratings. This study relied on the quasi-randomized allocation of patients to the wards, which either had a physical or VR calm room. Self-assessment scales (Montgomery-Åsberg Depression Rating Scale-Self Assessment [MADRS-S], Beck Anxiety Scale, and Clinical Global Impression) were used to determine the participants' baseline level of depressive and anxiety symptoms before their use of the physical or VR calm room. The study determined the state of well-being measured using an 11-point visual analog scale (VAS) as well as arousal measured by blood pressure (systolic and diastolic) and heart rate before and after the use of the calm rooms. The primary end point was self-reported well-being using the VAS.

**Results:**

A total of 60 participants were included—40 used the VR calm room and 20 used the physical calm room. The mean age of participants was 39 years and the majority were women (35/60, 58%). Analysis of VAS measurement showed improved well-being at the group level from before to after the intervention (*P*<.05), with no statistically significant difference in effects between the 2 different interventions. Effects were not moderated by baseline depression levels (dichotomized as MADRS-S >20 or ≤20) despite an overall difference in reported well-being between subgroups.

**Conclusions:**

Although the power in this study was low, the findings of this first study indicate comparable effects with respect to well-being and arousal of a VR calm room and a physical calm room. This suggests that a VR calm room can be a viable alternative when the use of a physical calm room is not an option for logistic or other reasons.

**Trial Registration:**

ClinicalTrials.gov NCT03918954; https://clinicaltrials.gov/ct2/show/NCT03918954

## Introduction

Interest in sensory rooms or so-called calm rooms in psychiatric inpatient care has increased significantly in recent decades. Originally developed for people with disabilities in the Netherlands during the 1970s [1], the purpose of sensory rooms is to offer patients a dedicated space for relaxation to improve their well-being and decrease their level of stress, anxiety, and distress—or even agitation and aggressive behavior [2-5]. This is typically achieved by providing soothing stimulation through sight, smell, hearing, touch, and taste [6]. It has been suggested that the implementation of sensory rooms has not only reduced stress but has also promoted overall safety in psychiatric inpatient units according to patient and staff experiences [7]. Sensory rooms can also be used as a tool to provide self-help and reduce distress through a convenient environment for the patients and, at the same time, strengthen the therapeutic relationship between the patient and the professional [6]. An important constraint for the use of sensory rooms, however, is that the resources required to fit the sensory room with appropriate elements and the fact that a dedicated room is required (entailing one less ward space), which may moreover be only used sporadically [8]. Furthermore, physical sensory rooms require oversight by a nurse to provide support, supervision, and ensure patient safety [6].

Instead of traditional relaxing techniques, innovative interventions with a high level of accessibility and effectiveness are needed [9]. Virtual reality (VR) is a powerful tool that promotes learning to benefit psychological well-being [10]. Furthermore, VR allows patients to immerse themselves in virtual environments adapted to their psychological state with safety [11]. In a previous study [12], participants were enthusiastic about using VR platforms in the future if they get access to VR technology.

VR is an immersive technology capable of creating a strong sense of presence in a simulated, virtual environment using stereoscopic displays and head tracking [13]. This, in turn, can be used for clinical purposes by creating virtual environments explicitly designed to promote mental well-being [14]. Studies have shown the potential for VR to treat mental conditions including social anxiety disorder [15,16], posttraumatic stress disorder [17], generalized anxiety disorder [18], and phobias [19,20]. As well as being used to treat or optimize different therapies, VR-based relaxation technology has also been used to provide immersive experiences for relaxation [21]. Also, various studies have shown that VR can induce well-being and lower stress levels [15,22-25]. Although most literature has focused on VR exposure therapy [20,26-28], VR-based relaxation is gaining increased research interest [21,23,29-31].

The VR relaxation setting provides a sense of presence, pleasure, activation, engagement, and a personalized experience [32]. VR relaxation typically involves situating the user in a soothing virtual nature environment that mirrors the use of nature scenery, for example, in physical calm rooms. Although there is some evidence that VR relaxation is efficacious in inducing relaxation, we are not aware of any previous studies that have evaluated VR relaxation as an alternative to having a physical sensory room in psychiatric inpatient care. Therefore, the aim of this study was to explore the effects of a VR calm room on self-reported well-being as well as blood pressure (BP) and heart rate (HR) compared to a physical calm room for patients admitted to psychiatric inpatient care.

## Methods

### Study Design

This quasi-randomized experimental study compared the effect of 2 types of calm rooms (physical and VR) on self-reported well-being and vital signs (BP and HR) pre- and postintervention among psychiatric inpatients. Patients who were already admitted were asked if they were interested in using a relaxation room and willing to provide ratings. They were included in the study if they fulfilled the inclusion criteria (see below) and provided written informed consent. Staff received instructions on how to use the calm rooms. As the 2 treatments were in different wards, it was not possible to allocate participants to each treatment arm in a truly randomized manner. The design was based on cluster-randomized design, but since there were only 2 units that participated, the study compares 2 arms for patients admitted to 2 comparable units.

### Study Population

Patients older than 18 years who were admitted to either of the 2 wards of psychiatric inpatient care at the Sahlgrenska University Hospital (Gothenburg, Sweden) were recruited. Exclusion criteria were diagnosis of paranoid schizophrenia or schizoaffective disorder; intellectual developmental disorder, organic brain injury, or other illness of significance likely to affect the patient’s decision-making skills; current abstinence symptoms from alcohol or other intoxicants; and any form of vision or balance disorder, manic episode, or suicidal thoughts.

### Study Setting

The study was conducted in 2 inpatient psychiatric wards specializing in bipolar disorder from March 2019 to February 2021. Although the wards specialized in bipolar disorder, they also received patients with other psychiatric diagnoses due to a lack of empty beds in other wards. Each ward had a responsible attending physician.

### Study Procedures

**Figure 1:**
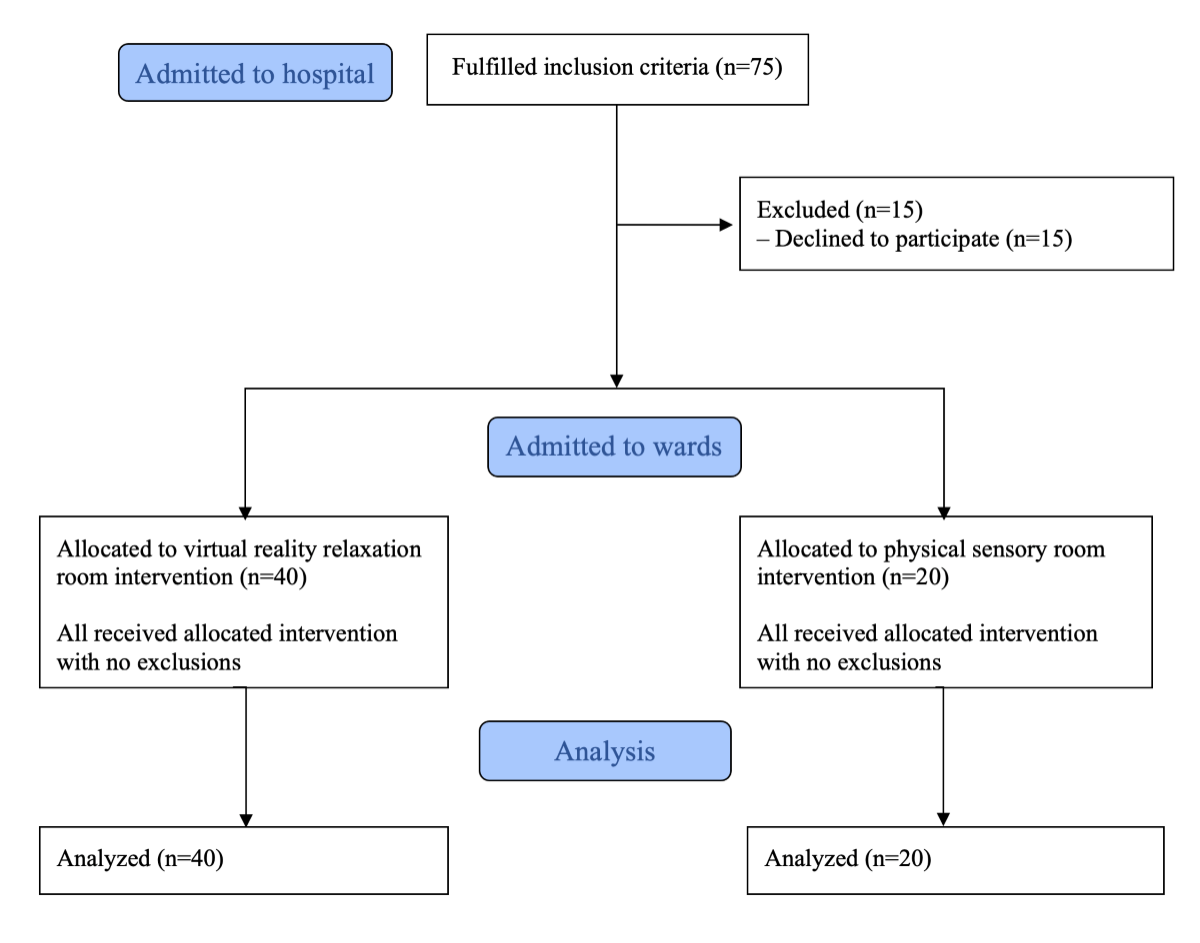
Flow diagram for the study participants.

Inpatients were informed about the study. In total, 75 patients fulfilled the inclusion criteria, of whom 15 declined to participate, leaving 60 patients who were included (Figure 1). After these patients had been given a detailed oral and written explanation of the study, all participants provided written informed consent. Patients were able to use the VR calm room (n=40) or physical calm room (n=20) in the respective inpatient wards to which they had been admitted. Participants who used the VR calm room received an introduction to the VR headset’s function and how to control the environment within the VR environment. Those who used the physical calm room received an introduction to the various sensory-stimulating elements in the room.

Two self-assessment scales (the Montgomery-Åsberg Depression Rating Scale-Self Assessment [MADRS-S] and the Beck Anxiety Inventory [BAI]) were used to establish the participants’ level of depressive and anxiety symptoms before their use of the VR or physical calm rooms. To evaluate the participants’ level of symptoms of depression and anxiety, 2 self-reported assessment scales were used as well as the clinical global impressions assessed by the staff.

Before the use of physical or VR calm room, staff measured each patient’s BP (systolic and diastolic) and HR (pulse). The patients were also asked to fill in a visual analog scale (VAS) covering their general level of well-being. BP and HR measurement and VAS assessment were repeated at the end of the session, with no missing data. The duration of stay in the calm rooms was determined either from the start of using the app in the VR calm room or from entering the door to the physical calm room until either exiting the app or the physical room.

The study was conducted in 2 different inpatient wards consisting of 14-15 beds for patients with acute psychiatric problems. In 2017, one of the wards introduced a physical calm room aimed at reducing stress and increasing psychological well-being. Currently, the other ward does not have a physical calm room. Therefore, the VR calm room consisting of a pair of wireless VR glasses was implemented in the second ward.

### Study Interventions

#### VR Calm Room

**Figure 2:**
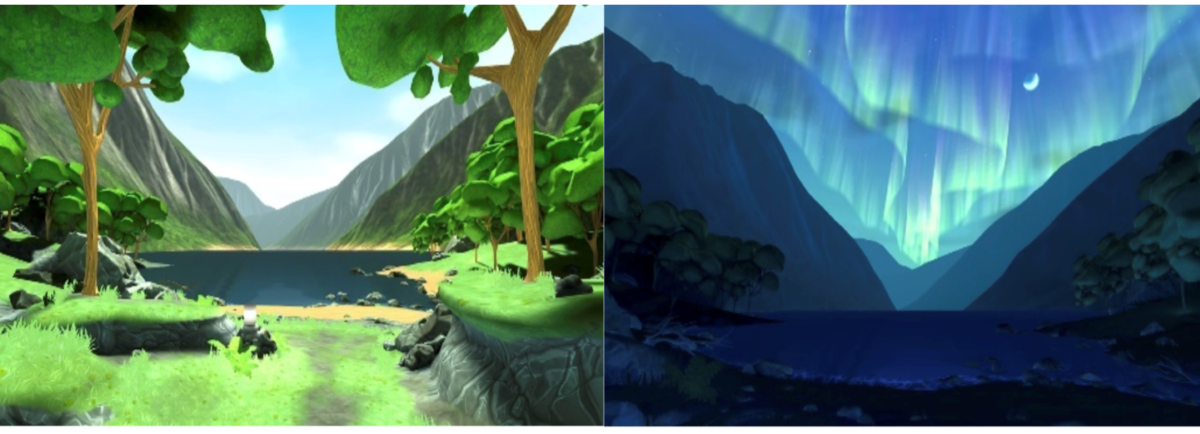
Screenshots from the virtual reality calm room in the application Calm Place, Mimerse.

To engage with the intervention, patients used a wireless, 3 degrees-of-freedom VR head-mounted display (an Oculus Go) running the Calm Place app developed by Mimerse. For example, screenshots are shown in Figure 2. The app offers a variety of soothing nature environments that can be tailored through, for example, day and night scenes and dynamic weather. In addition, the app includes breathing exercises, mindfulness programs, and relaxing music. The user can interact with the environment by looking at different objects inside the environment using the hand-held controller to adjust preferences and build individualized scenery. The app used includes virtual environments related to stress-reducing strategies and elements in virtual worlds [18,22].

The VR app is a real-time, 3D app developed in the game engine “Unity3D.” The VR app was also released on the Oculus store and was the highest rated app of its kind in the store. Similar to other VR relaxation or meditation apps, the user can choose in Calm Place to just sit and enjoy the gradual changes in nature, observing the change in light and sound, or to participate in guided relaxation meditation.

For the trial, a special version of the app was used, removing some customization (locked to 1 environment) and onboarding to make it easier to use in an academic setting. For instance, upon putting on the headset, the user would find themselves directly in the environment rather than having to navigate through a menu system to select from a list of environments.

During the trial, when the user puts on the headset and the app is loaded, the user finds themselves seated in a beautiful and stylized nature environment. In the environment, there is an object the user can point at and click to bring up a menu; however, many do not do this, simply staying and experiencing the default settings. VR training or previous experience is thus extremely low; it simply requires the user to put on the headset comfortably. Some choose to access and change the preferences from a menu that appears in the environment.

From the preferences menu, the user can set a time of day and freeze time (so that time of day would not gradually progress, the default setting). Each “full day” in the app takes a few minutes to complete. Time gradually shifts from morning to midday, to sunset, to calm night with northern lights, and then sunrise. From the preferences menu of the VR app, the user can also toggle through different types of weather such as rain, cloudy, or sunny. During rain, the environment and sound dramatically shift to a smooth drizzle and subdued colors. There was also an option to disable animals in the environment (birds in the distance, rabbits in the forest). Participants were in private rooms in the ward when using the VR glasses.

#### Physical Calm Room

**Figure 3:**
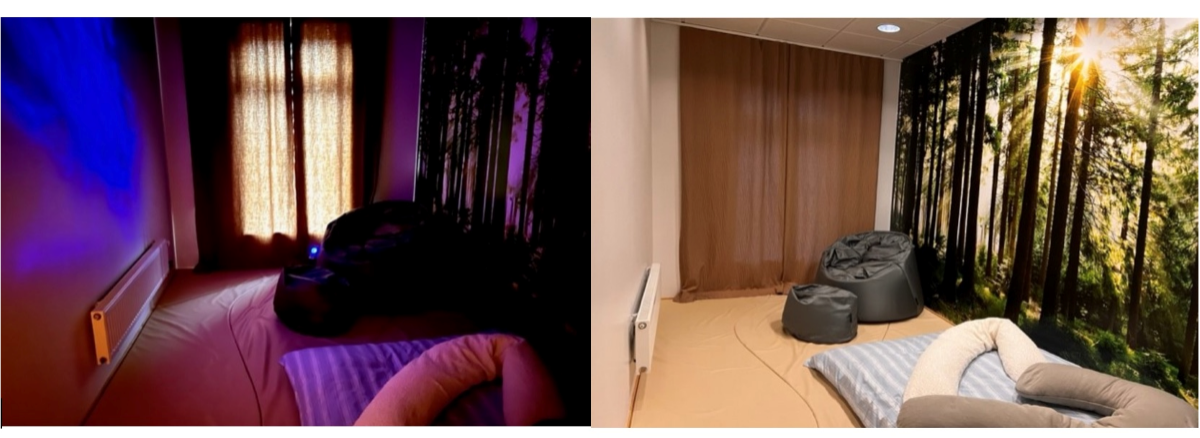
Photographs from the physical calm room.

Before study conception, a room in 1 of the wards was refitted to be a physical calm room (see photographs in Figure 3). This calm room was designed for active, relaxing stimuli in connection with vision, sensation, hearing, and smell. The room had a limited number of stimuli and all cognitively stimulating aspects, such as books, games, or other items were removed from the room. The following types of stimuli were available in the room:

1. Relaxation: the room’s floor is lined with a soft mattress that patients can lie on.
2. Vision: one of the walls has photo wallpaper that illustrates a forest, bringing the calming component of nature into the room. The other 3 walls are neutrally painted in a light pastel tone, giving a soothing sensation and time for processing the sensory impressions of the photo wallpaper. The room has large and luminous windows that are equipped with drapes. The room is also equipped with a projector that illuminates a starry sky on the ceiling and walls when the lights are turned off.
3. Haptic: a cover with balls is available in the room. There are sensory-stimulating balls and a sensory-stimulating mat with the same function that you can hold and also receive tactile massage.
4. Olfactory: fragrances may have a calming effect. Those who stay in this quiet room first receive sample dough and can then choose to bring a drop of ethereal aromatic oil made from lavender, lemon, refresh, or vanilla.
5. Auditory: there is an iPad in the room that can play a variety of relaxing music.

### Baseline Measurements

MADRS-S, a self-assessment tool for measuring depression symptoms, is used regularly in the clinic to evaluate depression symptoms. The scale contains 9 statements intended to measure various depression symptoms [33]. This scale was administered at the enrollment of the participants to intervention. BAI is a self-assessment instrument that measures the level of anxiety. The scale consists of 21 statements intended to measure different anxiety symptoms [34]. BAI was administered at the same time as MADRS-S. Clinical global impressions is a 7-point evaluation tool that requires the rater to determine the degree of the patient’s disease at the time of assessment in relation to the clinician’s previous experience in patients with the same diagnosis [35].

### Outcome Measurements

The primary clinical outcome changed in self-reported well-being state measured using the 11-point (0-10) VAS [36]. BP and HR were recorded through an electronic BP monitor before and after each session.

### Ethics Approval

Ethical approval was granted by the Swedish Ethical Review Authority (Dnr: 318-18). All participants provided written informed consent and could by choice end their use of the calm rooms at any time without specifying any reason. Patients at risk of self-harm or showing other signs that the use of a calm room might be harmful were not offered the use of the calm room.

### Statistical Analysis

The study originally aimed to include 72 patients where at least 36 would test each of the 2 calm rooms (36 in each arm) albeit not over 100 in total. However, due to COVID-19 restrictions and time constraints, the trial had to be ended prematurely when 60 patients had been included. The possibility of maintaining COVID-19 safety measures for the VR calm group meant that it was easier to recruit, resulting in more participants in that group.

Demographic and clinical characteristics of the patients were compared across groups using independent *t* tests and Fisher exact tests. Outcomes (VAS, BP, and HR) were analyzed using repeated measures ANOVA (RM-ANOVA) with standard time × group setups. Because there was no missing data, imputation was not necessary. In addition to the protocol, RM-ANOVA models were rerun as post hoc sensitivity analyses by also including baseline depression (dichotomized as MADRS-S >20 or ≤20) as an additional factor along with all possible interaction effects. Significant differences between groups were evaluated at *P*<.05 and, all analyses were performed using SPSS (version 27; IBM Corp).

## Results

The baseline demographic and clinical characteristics of all patients and by study arm are presented in Table 1. In total, the sample consisted of 58.3% (35/60) women and 41.7% (25/60) men. Mean age of the total study sample was 39.1 (range, 21-68) years. The groups were similar with respect to baseline characteristics, although there was a higher proportion of women in the physical calm room group (80.0%, 16/20 vs 47.5%, 19/40; *P*<.01). The mean duration of stay in the intervention was significantly longer for the physical calm room group compared to the VR calm room group (25.4 vs 15.2 minutes, *P*<.03).

The results of RM-ANOVA are summarized in Tables 2 and 3. There was a statistically significant effect of time on the VAS scale (*F*_1,58_=30.6, *P*<.01), with no interaction effects (*F*_1,58_*=*0.023, *P=*.80), suggesting no significant difference in relaxation effect between the 2 modalities. The same pattern was observed on HR. For systolic BP and diastolic BP, there were no effects of time. Sensitivity analyses revealed that in both groups, patients with a higher MADRS-S score had numerically lower score on the VAS scale compared to lower MADRS-S score both before and after the session (Tables 2 and 3). There were no 3-way interactions in the extended model, with the main effect of time on VAS ratings and HR remaining significant and no significant time by group effects. To control the sex difference between arms, these models were rerun with sex as an additional, fully factorial predictor—time by group findings remained unchanged and there was no time by group by sex interactions (results not shown).

**Table 1:** Demographic and clinical characteristics for all participants and by intervention type.

Characteristic					Total (N=60)		VR calm room (n=40)		Physical calm room (n=20)		*P* value			*t* test (*df*)	
Age (years), mean (SD)					39.1 (13.5)		40.7 (14.2)		35.8 (11.6)		0.28			N/A^a^	
**Gender, n (%)**												<.01			N/A
			Female	35 (58.3)		19 (47.5)		16 (80.0)					N/A		
			Male	25 (41.7)		21 (52.5)		4 (20.0)					N/A		
**Mental health diagnosis, n (%)**															
		Unipolar depression		18 (30.0)		10 (25.0)		8 (40.0)		.15			N/A		
		Anxiety disorder		13 (21.7)		10 (25.0)		3 (15.0)		.51			N/A		
		Bipolar disorder with depressive episode		12 (20.0)		9 (22.5)		3 (15.0)		.73			N/A		
		Bipolar disorder with hypomanic episode		13 (21.7)		10 (25.0)		3 (15.0)		.51			N/A		
		Bipolar disorder unspecified		4 (6.6)		1 (2.5)		3 (15.0)		.33			N/A		
**Baseline clinical measurement, mean (SD)**															
	MADRS-S^b^				22.4 (9.8)		22.2 (13.5)		22.7 (14.6)		.68			.118 (1,58)	
	Beck Anxiety Scale				20.2 (16.1)		22.5 (16.7)		15.5 (14.0)		.37			1.600 (1,58)	
	Clinical global impression				4.0 (0.8)		4.0 (0.8)		3.9 (0.9)		.27			.315 (1,58)	
Duration of stay in calm room (min), mean (SD)					18.6 (10.4)		15.2 (8.5)		25.4 (10.8)		.03			3.973 (1,58)	

^a^N/A: not applicable.

^b^MADRS-S: Montgomery-Åsberg Depression Rating Scale-Self Assessment.

**Table 2:** Repeated measures ANOVA before and after the intervention within and between subjects: model 1.

Variable	VR calm room, mean (SD)			Physical calm room, mean (SD)					*F* test (*df*; *P* value^a^)												
	Preintervention	Postintervention	Preintervention		Postintervention			T^b^			G^c^		TxG		M^d^		MxT		MxG		TxGxM
VAS^e^	5.2 (2.3)	6.5 (2.6)	4.5 (2.3)			5.9 (2.8)		30.6 (1,58; *<.01*)			0.9 (1,58; .30)		0.02 (1,58; .80)		—^f^		—		—		—
SBP^g^	128 (16)	124 (15)	116 (10)			116 (10)		1.9 (1,58; .10)			7.8 (1,58; *.007*)		1.8 (1,58; .10)		—		—		—		—
DBP^h^	73 (10)	73 (13)	68 (12)			67 (9)		0.3 (1,58; .50)			3.3 (1,58; .07)		0.3 (1,58; .50)		—		—		—		—
HR^i^	85 (15)	81 (15)	76 (10)			75 (10)		5.2 (1,58; *.02*)			3.9 (1,58; .50)		1.1 (1,58; .20)		—		—		—		—

^a^Italicized values are significant at *P*<.05.

^b^T: time.

^c^G: group.

^d^M: Montgomery-Åsberg Depression Rating Scale-Self Assessment (MADRS-S).

^e^VAS: Visual Analog Scale.

^f^Not available.

^g^SBP: systolic blood pressure.

^h^DBP: diastolic blood pressure.

^i^HR: heart rate.

**Table 3:** Repeated measures ANOVA before and after the intervention within and between subjects: model 2.

Variable	VR calm room, mean (SD)					Physical calm room, mean (SD)					*F* test (*df*; *P* value^a^)						
	M_H_^b^	M_L_^c^	M_H_	M_L_	M_H_		M_L_	M_H_	M_L_	T^d^		G^e^	TxG	M^f^	MxT	MxG	TxGxM
VAS^g^	4.0 (2.0)	6.8 (1.7)	5.2 (2.1)	8.3 (2.8)	3.1 (1.3)		4.7 (2.7)	6.3 (2.1)	7.4 (2.2)	29.2 (1,56; *<.01*)		1.9 (1,56; .10)	<0.01 (1,56; .90)	33.6 (1,56; *<.01*)	0.7 (1,56; .70)	0.001 (1,56; .90)	0.6 (1,56; .40)
SBP^h^	127 (18)	129 (12)	125 (14)	123 (16)	119 (9)		113 (11)	119 (10)	112 (9)	2.3 (1,56; .10)		8.2 (1,56; *.006*)	2.0 (1,56; .10)	0.7 (1,56; .30)	1.1 (1,56; .30)	0.9 (1,56; .30)	0.3 (1,56; .50)
DBP^i^	71 (12)	74 (8)	73 (16)	72 (9)	69 (11)		68 (14)	68 (10)	66 (7)	0.5 (1,56; .40)		3.3 (1,56; .07)	0.2 (1,56; .60)	0.10 (1,56; .90)	0.9 (1,56; .30)	0.1 (1,56; .70)	0.2 (1,56; .60)
HR^j^	83 (15)	87 (17)	81 (15)	82 (16)	75 (8)		78 (13)	74.5 (9)	76 (10)	6.01 1,56; *.02*)		3.8 (1,56; .05)	1.3 (1,56; .20)	0.4 (1,56; .50)	1.5 (1,56; .20)	0.002 (1,56; .90)	0.3 (1,56; .50)

^a^Italicized values are significant at *P*<.05.

^b^M_H_: high MADRS-S >20.

^c^M_L_: low MADRS-S ≤20.

^d^T: time.

^e^G: group.

^f^M: Montgomery-Åsberg Depression Rating Scale-Self Assessment (MADRS-S).

^g^VAS: Visual Analog Scale.

^h^SBP: systolic blood pressure.

^i^DBP: diastolic blood pressure.

^j^HR: heart rate.

## Discussion

### Overview

The main objective of this quasi-randomized study was to explore the effects of a physical calm room on self-reported well-being compared to a VR calm room for patients admitted to inpatient psychiatric intensive care. We did not find any statistically significant between-group differences in relaxation effects. However, both groups reported increased well-being and decreased HR, albeit with no effect on BP. Effects were not moderated by baseline depression levels despite an overall difference in reported well-being between subgroups.

To our knowledge, this is the first study to compare the effects of a physical calm room to VR relaxation. Our findings are, however, in line with previous studies on both physical calm rooms [1,3,5] and VR relaxation [23,25,31,37]. Our results suggest that VR relaxation can be considered when there is no opportunity to have a physical calm room due to limitations of space, safety reasons, financial limitations, or similar. Designed VR environments used in clinical therapies, such as exposure therapies for phobias, can resolve many logistic issues [28,29] and support patient autonomy. In addition, VR supports the ability to relax by increasing the state of mindful awareness [38]. Both VR apps and physical calm rooms have shown an increased sense of “relaxation” [6]. In our study, participants reported that overall well-being was significantly increased.

Relaxation is the state of calmness that fosters positive well-being including strategies, such as breathing exercises, meditation, and guided muscle relaxation. With these strategies, high BP and HR may be lowered [9]. Effects of VR calming rooms are not explored, but there is a couple of studies that show reduced distress levels and lower HR [39,40]. A previous study [41] supports that HR and some other physical signs of arousal reduced after the use of multisensory treatment as well that patients felt more relaxed, focused, calm, and comfortable aligned with the results of our study and allows to draw conclusion that sensory interventions are likely to reduce distress inducing the subjective well-being in psychiatric inpatient care.

Limitations of this study include lower than the planned statistical power, as well as the quasi-random allocation to the different calm room interventions. According to the initial trial design, the sample was set to include 100 participants with a diagnosis of bipolar disorder. Due to the COVID-19 pandemic, the study was paused between January 2020 and July 2020, making it unfeasible to recruit the target sample size during the study timeframe. In addition, the hospital had a lack of beds, and patients who were diagnosed with other than bipolar disorder were included in the study. The achieved sample size of 60 (40+20) allowed us to detect with 80% power, a between-group difference of d>0.78 postintervention compared to d>0.57 as originally planned. Moreover, the lack of a predefined, clinically anchored, noninferiority limit precludes us from analyzing the study using this approach. Regarding the quasi-random allocation, this was a necessity stemming from running the study at a psychiatric inpatient clinic. Allocation, in this fully naturalistic trial, was based by necessity on clinic prerequisites (eg, bed availability and staffing) and not on study-specific allocation; thus, because it was not fully randomized, allocation was nonetheless seemingly independent of participant factors that could risk confounding results. For example, patients were not allocated to the different wards (and therefore interventions) due to severity of distress.

Difference in achieved sample size ratio, as well as some differences in patient characteristics and baseline measures, indicate that the allocation was not completely free of systematic influences. Moreover, almost all the physical calm room group were female (16/29, 80%) compared to an even gender distribution in the VR room group. Furthermore, patients have not had any option to choose the room they wanted to experience, but they were allocated to the respective wards. The gender imbalance considers as a limitation because we cannot report if the intervention equal associated with an induced well-being because of gender as a factor. We may assume that the gender imbalance exists toward the physical sensory room due to different preferences between women and men. Alternatively, supervising personal may assume that women prefer a sensory room. The personal have had the responsibility to check the inclusion and exclusion criteria as well as the clinical characteristics and may have excluded more male patients than females or became influenced by social norms which indicate that males not seeking help [42].

In addition, length of stay was determined for the 2 calm rooms; however, this should not be considered an outcome in itself. The importance of measuring time spent in the intervention is hard to interpret in a quantitative study and should rather be explored in a qualitative way. A previous study shows that the greater amount of time spent in sensory room did not necessarily related with stress-relieving outcomes [43]. A further limitation is that the calm modalities of VR and a physical calm room are not the same; however, the comparison of using these 2 methods for relaxation should not be affected in a serious manner.

Future research could examine whether VR relaxation can decrease the use of sedatives as well as aggressive behavior in the ward. Some studies have found no link between seclusion and aggressive behavior with the use of physical calm rooms. One small study did not find a decreased frequency of self-harm during the test period, although there was a low level of participation [2]. The same applies to another small study that did not find a reduction in the rate of seclusion for 13 participants [44]. The wards included in our study primarily receive patients with a diagnosis of bipolar disorder. Therefore, when the study was planned, patients with schizophrenia were excluded due to the practical reason of them being very few in these units. However, patients with a psychotic diagnosis need to be included in future studies. Due to small sample size and lack of controlled trials, research on physical calm rooms remains inconclusive, albeit promising. However, VR technology has become affordable and more accessible, which has led to its increasingly widespread use in psychiatric care [45].

### Conclusions

In conclusion, both VR and physical calm rooms were associated with an improvement in overall well-being and appear to be feasible self-relaxation methods for use in psychiatric inpatient care. Furthermore, no significant differences were found between the 2 different interventions, although power was low in this first study of a VR calm room in psychiatric inpatient setting.

## Abbreviations

BAI: Beck Anxiety Inventory
BP: blood pressure
HR: heart rate
MADRS-S: Montgomery-Åsberg Depression Self-Rating Scale
RM-ANOVA: repeated measures ANOVA
VAS: visual analogue scale
VR: virtual reality

## Data Availability

Due to local regulations, we are not able to share the data freely but raw data can be provided from the corresponding author on reasonable request.
